# Municipal Solid Waste Landfills Harbor Distinct Microbiomes

**DOI:** 10.3389/fmicb.2016.00534

**Published:** 2016-04-20

**Authors:** Blake W. Stamps, Christopher N. Lyles, Joseph M. Suflita, Jason R. Masoner, Isabelle M. Cozzarelli, Dana W. Kolpin, Bradley S. Stevenson

**Affiliations:** ^1^Department of Microbiology and Plant Biology, University of OklahomaNorman, OK; ^2^U.S. Geological SurveyOklahoma City, OK, USA; ^3^U.S. Geological SurveyReston, VA, USA; ^4^U.S. Geological SurveyIowa, IA, USA

**Keywords:** landfill, leachate, microbiome, microbial ecology, chemicals of emerging concern

## Abstract

Landfills are the final repository for most of the discarded material from human society and its “built environments.” Microorganisms subsequently degrade this discarded material in the landfill, releasing gases (largely CH_4_ and CO_2_) and a complex mixture of soluble chemical compounds in leachate. Characterization of “landfill microbiomes” and their comparison across several landfills should allow the identification of environmental or operational properties that influence the composition of these microbiomes and potentially their biodegradation capabilities. To this end, the composition of landfill microbiomes was characterized as part of an ongoing USGS national survey studying the chemical composition of leachates from 19 non-hazardous landfills across 16 states in the continental U.S. The landfills varied in parameters such as size, waste composition, management strategy, geography, and climate zone. The diversity and composition of bacterial and archaeal populations in leachate samples were characterized by 16S rRNA gene sequence analysis, and compared against a variety of physical and chemical parameters in an attempt to identify their impact on selection. Members of the Epsilonproteobacteria, Gammaproteobacteria, Clostridia, and candidate division OP3 were the most abundant. The distribution of the observed phylogenetic diversity could best be explained by a combination of variables and was correlated most strongly with the concentrations of chloride and barium, rate of evapotranspiration, age of waste, and the number of detected household chemicals. This study illustrates how leachate microbiomes are distinct from those of other natural or built environments, and sheds light on the major selective forces responsible for this microbial diversity.

## Introduction

The global upsurge in urbanization of the human population is associated with even greater increases in the generation of municipal solid waste (MSW). By the year 2025, 4.3 billion urban residents are projected to generate approximately 1.42 kg of MSW per person, totaling 6.1 million metric tons per day, making the generation of MSW an even faster growing pollutant than greenhouse gases (Hoornweg et al., 2013). Advances in waste reduction, recycling, and composting have made an impact on the fate of MSW, but landfilling is still the most common waste disposal option and is likely to remain so for the foreseeable future. Despite the heavy reliance on this method of disposal, surprisingly little is known about the microbiology and its function in these engineered ecosystems.

The degradation of organic matter in landfills is broadly characterized by a succession of phases that ultimately result in the conversion of the waste materials to mineralized end products like water, CO_2_, and CH_4_ (Palmisano and Barlaz, 1996). Complex assemblages of bacteria and archaea carry out the majority of MSW degradation. These syntrophic consortia are far more capable of mineralizing the myriad of organic substances deposited in landfills than single microorganisms or populations. Both chemical profiles and microbial community composition change during the biodegradation of MSW, but the general patterns are consistent with the anaerobic cycling of organic matter (McInerney et al., 2009). In addition to the resident bacterial and archaeal populations, landfill leachates are also home to populations of anaerobic fungi. Previously members of the order Neocallimastigales, known to degrade cellulose, were found in British landfill leachates (Lockhart et al., 2006). The accumulation of organic acids can decrease the pH of the landfill, occasionally inhibiting the overall degradation processes (Mormile et al., 1996). The accumulation of acid is transient and modulated by the subsequent metabolism of organic acid intermediates, which returns the pH of a landfill to near-neutral values that are conducive to methanogenesis (Barlaz et al., 1989). An additional factor related to microbial metabolic activity in landfills is the moisture content of the refuse material (Suflita et al., 1992; Gurijala and Suflita, 1993). The shifting, heterogeneous physical and chemical profiles of landfills are certainly a main reason why they are home to such a diverse assemblage of microorganisms exhibiting a broad range of metabolic activities (Mori et al., 2003; Gomez et al., 2011; Lu et al., 2012).

The materials deposited in landfills are the sum total of numerous human activities, chemically and physically diverse, and challenging to fully degrade. The incomplete degradation of MSW leads to the production of leachate that can solubilize many chemicals of emerging concern (CECs; Eggen et al., 2010). These CECs include a complex range of chemicals found in household, commercial, and industrial products, whose impact on the environment and human health are not well-understood (Palm et al., 2002; Pal et al., 2010; Andrews et al., 2012; Masoner et al., 2016). The interaction between the microbial community, leachate, and CECs in landfills is of great interest. Previous studies have suggested that a number of genes associated with the degradation of CECs are present within landfills (Lu et al., 2012). Specifically, it is of interest to know if the complex chemistry of landfill leachate enriches or selects for particular types of microorganisms that might be capable of metabolizing such compounds. As an important step in exploring such potential interactions, we conducted *post-hoc* tests for the potential correlation between leachate microbiota and the presence of numerous CECs.

The microbial assemblages associated with leachate samples from 19 landfills were characterized using high-throughput sequencing of 16S rRNA gene libraries. Species richness, evenness, and shared diversity were compared between each sample. We investigated the connection between microbial communities in landfill leachates and several operational and environmental variables, as part of a broader study (Masoner et al., 2014). The *a priori* predictions that geographic region, waste profiles, geology, or annual rainfall would impact the composition of the microbial community were tested. Correlations between the microbial communities and landfill management characteristics such as leachate produced per year, waste dissolution time, the amount of waste accepted per year, and the age of the landfills, also were tested *ad hoc*. While many previous studies have investigated the microbiology of landfills (McDonald et al., 2010; Mouser et al., 2010; Lu et al., 2012), this study surveyed a larger number of landfill leachate samples across the United States using a sequencing-based approach. Therefore, the present study represents a more comprehensive analysis of microbial diversity to characterize the “landfill microbiome” and the selective forces responsible for its formation.

## Materials and methods

### Sample collection

**Figure 1:**
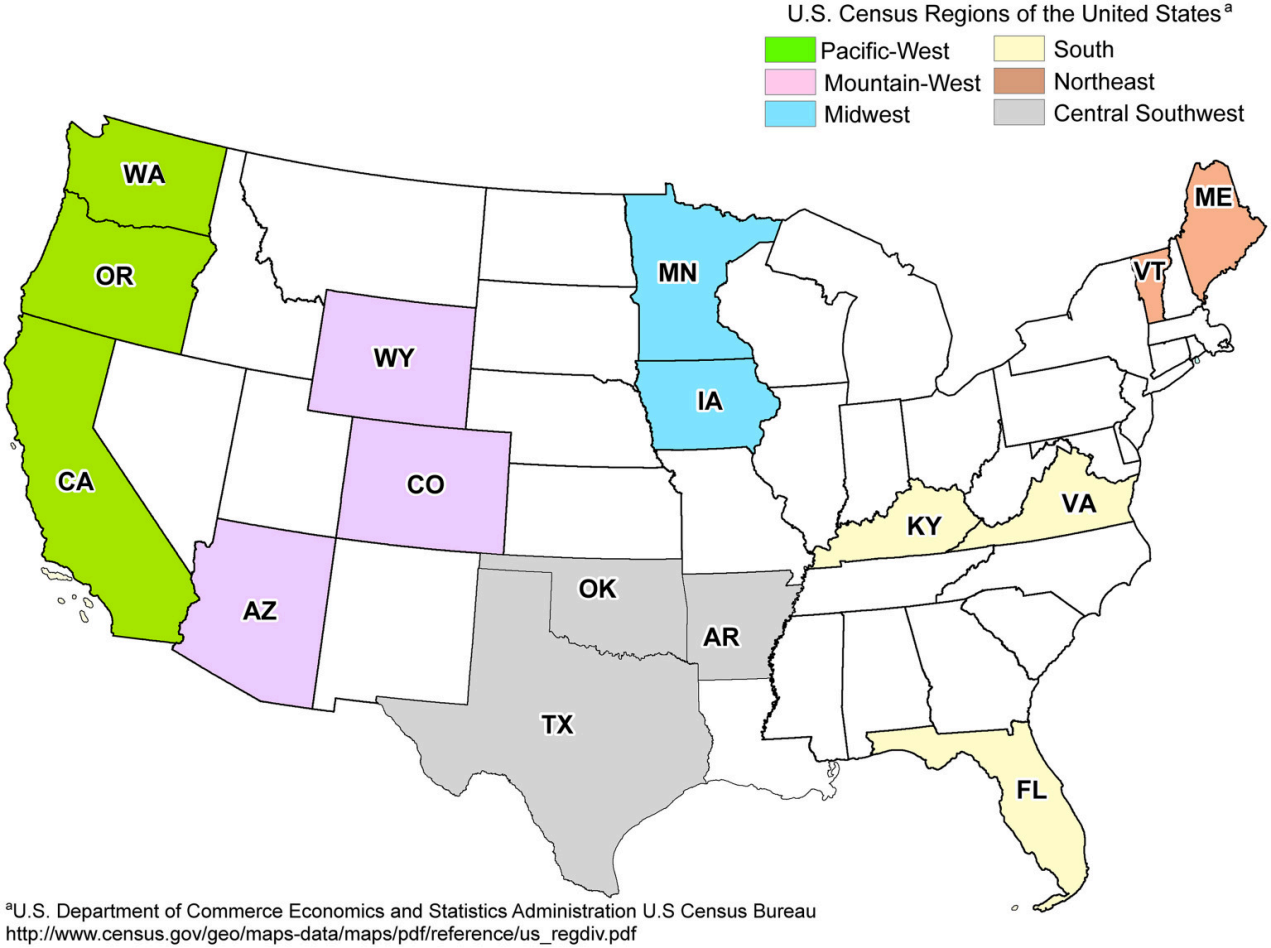
**Map of landfill sites sampled across regions of the United States**. States containing sampled landfills are labeled in bold. One landfill was sampled per state, with the exception of OK (2) and ME (3). Adapted from Masoner et al. (2014).

Fresh leachate samples were collected from 19 landfills from six different regions across the United States in triplicate (57 total samples) during the summer and fall of 2011 by on-site technicians (Figure 1). The metadata used in this study were also used to determine the potential impact of environmental parameters on the distribution of CECs detected within landfills (Masoner et al., 2014). Any pipelines or tubing used to collect leachate were purged with at least three volumes or for 5 min to remove stagnant leachate or other contaminants. All equipment and tubing were field rinsed with at least 1 L of leachate prior to sampling. Triplicate samples of biomass and particulate matter were collected through a sterile in-line polypropylene filter (Advantec; 47 mm diameter) pre-loaded with a nitrocellulose membrane filter (Whatman; 0.45 μm pore size, 47 mm diameter). Volumes of leachate filtered varied from 8 to 1500 mL at the discretion of the on-site technicians, based on restriction of flow due to filter obstruction (Table S1). After filtration, the nitrocellulose filter was removed from the holder using sterile forceps, transferred to 5 mL of DNAzol (Molecular Research Center, Inc., Cincinnati, OH, USA), shipped overnight to the University of Oklahoma, and stored at −80°C until DNA extraction.

### DNA extraction and 16S rRNA gene library preparation

Each sample (filter and DNAzol) was vortexed for 30 s at full speed upon thawing at room temperature. DNA was extracted from 1 mL of the DNAzol solution in each replicate sample using an automated Maxwell 16 Cell total RNA LEV Purification Kit (Promega, Madison, WI, USA), omitting the final DNase step as described in Oldham et al. (2012). Extracted DNA ranged in detected concentration from 1.04 to 12.3 ng/μL, with three samples that failed to quantify (Table S1). Libraries of bacterial and archaeal 16S rRNA gene fragments were amplified from each DNA extract using PCR with primers that spanned the V4 region between position 519 and 802 (*E. coli* numbering), producing a ~300 bp fragment. These primers evenly represent a broad distribution of both the Bacteria and Archaea (Klindworth et al., 2013). The forward primer (M13L-519F: 5′- **GTA AAA CGA CGG CCA G**CA CMG CCG CGG TAA -3′) contains the M13 forward primer (in bold), followed by the 16S rRNA gene-specific sequence (underlined). The reverse primer (785R: 5′-TAC NVG GGT ATC TAA TCC-3′) was taken directly from the reverse primer “S-D-Bact07850b-A-18” in Klindworth et al. (2013). Each 50 μL PCR consisted of 1X DreamTaq PCR master mix (ThermoFisher Scientific, Waltham, MA, USA), 0.1 μM of each primer, and 5–10 μL of 1:10 dilutions of DNA extracts. Additional details of the PCR are provided in the file Supplementary Information. The amplified 16S rRNA gene fragments in each library were purified using the Wizard SV Gel and PCR Clean-Up System (Promega, Madison, WI, USA) according to manufacturer's protocols. A second, six cycle PCR was used to add a unique 12 bp barcode (Hamady et al., 2008) to each amplicon library using a forward primer containing the barcode+M13 forward sequence (5′-3′) and the 785R primer [See the file Supplementary Information]. The resulting barcoded PCR products were quantified using the QuBit HS assay (Life Technologies, Carlsbad, CA, USA), pooled in equimolar amounts, and concentrated to a final volume of 80 μL using two Amicon® Ultra-0.5 mL 30K Centrifugal Filters (Millipore). The final pooled library was then submitted for sequencing on the MiSeq platform using PE250 V2 chemistry (Illumina, San Diego, CA, USA).

### Sequence analysis

After sequencing, reads were merged using PEAR (Zhang et al., 2014), demultiplexed in QIIME (Caporaso et al., 2010b), filtered by quality, and clustered into operational taxonomic units (OTUs) using UPARSE (Edgar, 2013). Taxonomy of each OTU was assigned using UCLUST (Edgar, 2010) and the SILVA database (Release 119; Pruesse et al., 2007). A representative sequence of each OTU was aligned with pyNAST (Caporaso et al., 2010a) against an aligned version of the SILVA r119 database, and filtered to remove uninformative bases. A phylogenetic tree was generated using the maximum likelihood method and a Jukes Cantor evolution model within FastTree (Price et al., 2010), and used for community composition analyses. Multiple diversity metrics including abundance-based coverage estimation [ACE; (Magurran, 2013)], the number of observed OTUs, and the Shannon equitability index, which is the inverse of the Shannon index H (*H* = $\overset{S_{obs}}{\underset{i=1}{\sum }}\frac{n_{i}}{N}ln\frac{n_{i}}{N}$) (Shannon, 1948), were used at a normalized sequence depth (*n* = 6000). Differences in community composition were estimated using weighted and unweighted UniFrac indices (Lozupone and Knight, 2005). A tree comparing samples was generated with the Unweighted Pair Group Method with Arithmetic Mean (UPGMA) method based on a jackknifed distance matrix of the weighted UniFrac index. Each library was subsampled to 6000 reads to generate a weighted UniFrac distance matrix for comparison among landfill leachate samples, and 300 reads to generate an unweighted UniFrac distance matrix for the meta-analysis of multiple microbial communities. A core microbiome was computed for all samples within QIIME. A mapping file is included as Table S2, and the commands used to produce the final BIOM file are publicly available at http://dx.doi.org/10.5281/zenodo.15665. Unclassified OTUs were identified during analysis in QIIME. Representative sequences aligned to the latest SILVA database, were added to the non-redundant tree (SILVA r123 NR99) within the phylogenetic software package ARB (Ludwig et al., 2004) in order to approximate taxonomy based on phylogeny. Closely related sequences were marked and retained with the unclassified OTU sequences to form the phylogenetic tree in Figure S1.

### Meta-analysis of microbial communities

To compare the microbial assemblages in landfill leachate to those in other environments, datasets representing a broad diversity of environments were obtained from qiita.microbio.me. These studies represented diverse environments including sediments, soils (QIITA Study 619), saline and fresh waters (Caporaso et al., 2011), bog and permafrost soils (QIITA Study 1036), contaminated sediments and waters (QIITA Studies 1039 and 1197), sediments near the Deepwater Horizon Oil Spill (QIITA Study 1198), waste water treatment plant effluent, and human and canine associated microbiomes (Caporaso et al., 2012). A QIIME compatible mapping file of the samples used for the meta-analysis is included in Table S3. Closed reference OTU picking was required for the meta-analysis because different primers were used to generate the 16S rRNA gene fragments in each of these libraries. The OTUs were clustered using UCLUST (Edgar, 2010) and the SILVA database (release 119) as a reference, which itself was clustered at 97% sequence similarity. An even sampling depth of 300 sequences per sample was chosen for ordination of all samples using an unweighted UniFrac distance matrix.

### Statistical analyses

To assess the significance of *a priori* predictions, a non-parametric permutational multivariate analysis of variance (PERMANOVA) test was run within QIIME using the adonis method. All PERMANOVA analyses used the weighted UniFrac distance matrix as the community matrix input, which was then compared against categorical and continuous environmental variables. Multiple PERMANOVA analyses were run for *post-hoc* testing of environmental variables (Table S4) and CECs (Table S5). Given the large number of variables tested, *p*-values were adjusted with the false discovery rate (FDR) method (Benjamini and Yekutieli, 2001) using the command p.adjust in R. All other statistical analyses were performed within QIIME using the R package “vegan” (Dixon, 2003). As a final exploratory method for correlations not assumed *a priori*, the Bio-Env method (Clarke and Ainsworth, 1993) was used to identify combinations of continuous environmental parameters that were best correlated with community composition. Iterative comparisons between the weighted UniFrac distance matrix and the Euclidian distances generated by selected physiochemical categories listed in Table S6 were used to identify the subset of selected categories that best explains the variation in the distance matrix describing the microbial assemblages in landfill leachates.

### Data availability

After sequencing, raw reads were deposited in the NCBI sequence read archive (SRA) under the accession number SRX864556 (http://www.ncbi.nlm.nih.gov/sra). Raw sequencing reads generated in this study used for meta-analyses are deposited in the SRA under the accession number SRX1629938. The mapping file used to generate the BIOM file needed for figure generation is given in Table S2.

## Results

**Figure 2:**
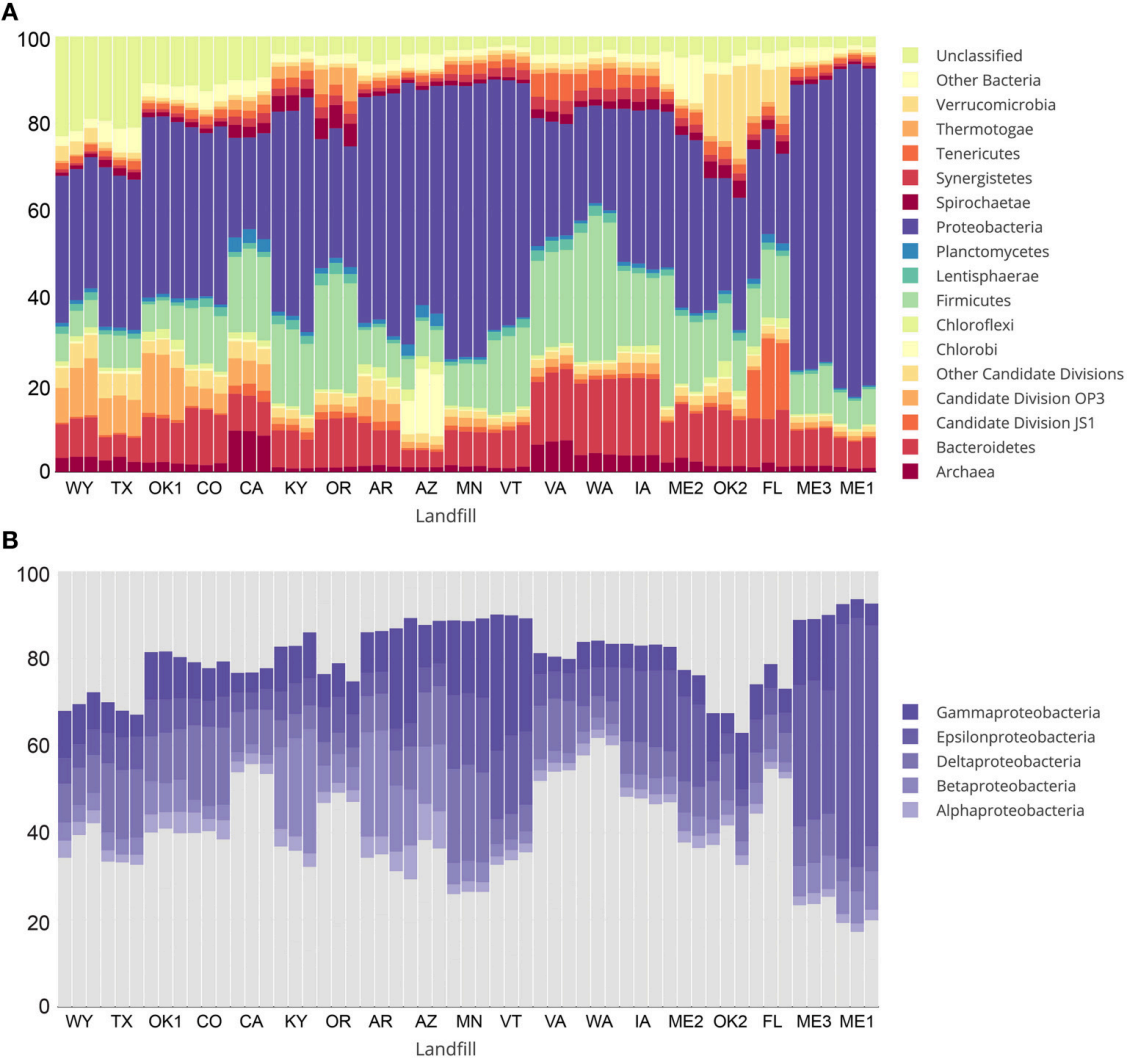
**Taxonomic summary showing percent relative abundance of bacterial and archaeal phyla from sampled landfill leachates are shown in (A)**. Taxa representing less than an average of 1% relative abundance are grouped together as “Other Bacteria” or “Other candidate divisions” for clarity. Relative abundance of classes within the most abundant phylum, Proteobacteria, are shown in **(B)**.

Over 1 million high quality reads from triplicate leachate samples of 19 landfills were retained after processing for quality and removal of chimeric sequences. A total of 4987 OTUs were detected among all samples, representing a broad taxonomic diversity at 97% sequence similarity. More than 10% of all OTUs at CO, CA, OK1, TX, and WY were designated as “unclassified” (Figure 2A). The largest unclassified OTU, OTU2 was most closely related to mitochondrial 16S rRNA gene sequences from the eukaryotic fungal-like Oomycetes. All other “unclassified” OTUs represented a broad phylogenetic distribution (Figure S1) and were present in low abundance in any leachate sample.

The most abundant taxa across all landfill leachates were numerous lineages of the Proteobacteria, including the Beta-, Delta-, Epsilon-, and Gamma-proteobacteria. Alphaproteobacteria were detected in relative abundances below 5% in all tested landfills (Figure 2B). The abundance of the Proteobacteria differed between groups of landfills, with the Deltaproteobacteria being the most abundant at TX, OK1, CO, CA, and MN. The abundance of Deltaproteobacteria was distributed between numerous lineages, with the Syntrophobacterales, Desulfuromonadales, and Desulfovibrionales being the most abundant. The landfill leachates from ME1, ME2, ME3, MN, VT, and IA contained the highest relative abundance of Epsilonproteobacteria (>15%), which were composed almost entirely of members of the order Campylobacterales (Figure 2B). The Betaproteobacteria were present across all landfills, yet they were the most abundant at KY, AR, and AZ (>10%), and primarily were composed of members of the order Burkholderiales. An exception was at AZ, where members of the order Hydrogenophilales were the most abundant Betaproteobacteria. The Gammaproteobacteria were present in all landfills, with the highest abundance observed at AR, AZ, ME3, MN, and VT. The order Pseudomonadales was the most abundant across almost all landfills, although OK1 contained a population of Methylococcales and a correspondingly lower relative abundance of the Pseudomonadales. The Bacteroidetes and Firmicutes composed the next two most abundant phyla at almost all landfills, and were dominated by the Bacteroidales and Clostridiales. Relative abundances of detected OTUs can be found in Table S8, and the absolute abundances of each OTU can be found in Table S9.

Members of the Archaea were notably low in relative abundance, ranging from 0.8 to 4.35% across most landfills, with the exception of CA and VA, which contained between 6.28 and 9.33% Euryarchaeota (Figure 2A). No Crenarchaea were detected, and the Thaumarchaeota ranged from 0 to 0.03% in any landfill. The OTUs most closely related to methanogenic archaea were detected at the highest abundance in WA and VA. Each contained a different assemblage of methanogens, with WA primarily containing members of the order Methanobacteriales, with the most abundant OTU (1.62 to 2.16%) related to the genus *Methanothermobacter*. The landfill in VA, however, contained an OTU most closely related to the candidate genus “*Methanomethylophilus*,” within the order Thermoplasmatales. Interestingly, this candidate genus was also recently detected in a directed search for archaea within a landfill in India (Yadav et al., 2015). These close relatives of the genus “*Methanomethylophilus*” were the most abundant methanogens detected across any sample, with a relative abundance of 4.58–5.58% within the VA landfill.

The species richness in each landfill leachate sample was expressed as the number of observed OTUs as a function of sample read depth in Table 1. Specific diversity values for each landfill are included in Table S7. In addition to high species richness, the relative abundance of OTUs present in each landfill leachate sample was distributed rather evenly, as indicated by Shannon's equitability values (*E*_*H*_) above 0.700 for all samples with the exception of ME1. Samples from ME1 were the least even (median *E*_*H*_ of 0.643), which could be explained by the high relative abundance of Epsilonproteobacteria (Figure 2B). The abundant Epsilonproteobacterial OTUs in ME1 were members of the order Campylobacterales and closely related to the genus *Arcobacter* (Table S8). Because of the enrichment of the Campylobacterales, samples from ME1 were considered outliers and excluded from further ordination or analysis. Of the 4987 OTUs detected across all landfill leachates, only 147 (2.9% of all detected OTUs) were shared across all landfills. This core microbiome, however, represented 49% of all sequence reads in the study and represented the broad abundant taxonomic lineages detected in the landfill survey (Table S10).

**Table 1:** **Summary of diversity indices^a^ across all tested landfill leachates**.

**Metric**	**Min**	**Max**	**Median**
ACE	1776.077	2511.951	2172.765
PD	20.725	30.400	25.090
Observed OTUs	874.90	1396.50	1181.15
*E_*H*_*	0.612	0.8856	0.817

a Values given represent minimum, maximum, and median values for abundance-based coverage estimation (ACE), Faith's Phylogenetic Diversity (PD), number of observed OTUs (Obs OTUs), and Shannon's equitability index values (E_H_). A more detailed summary of diversity indices is available as Table S7.

**Figure 3:**
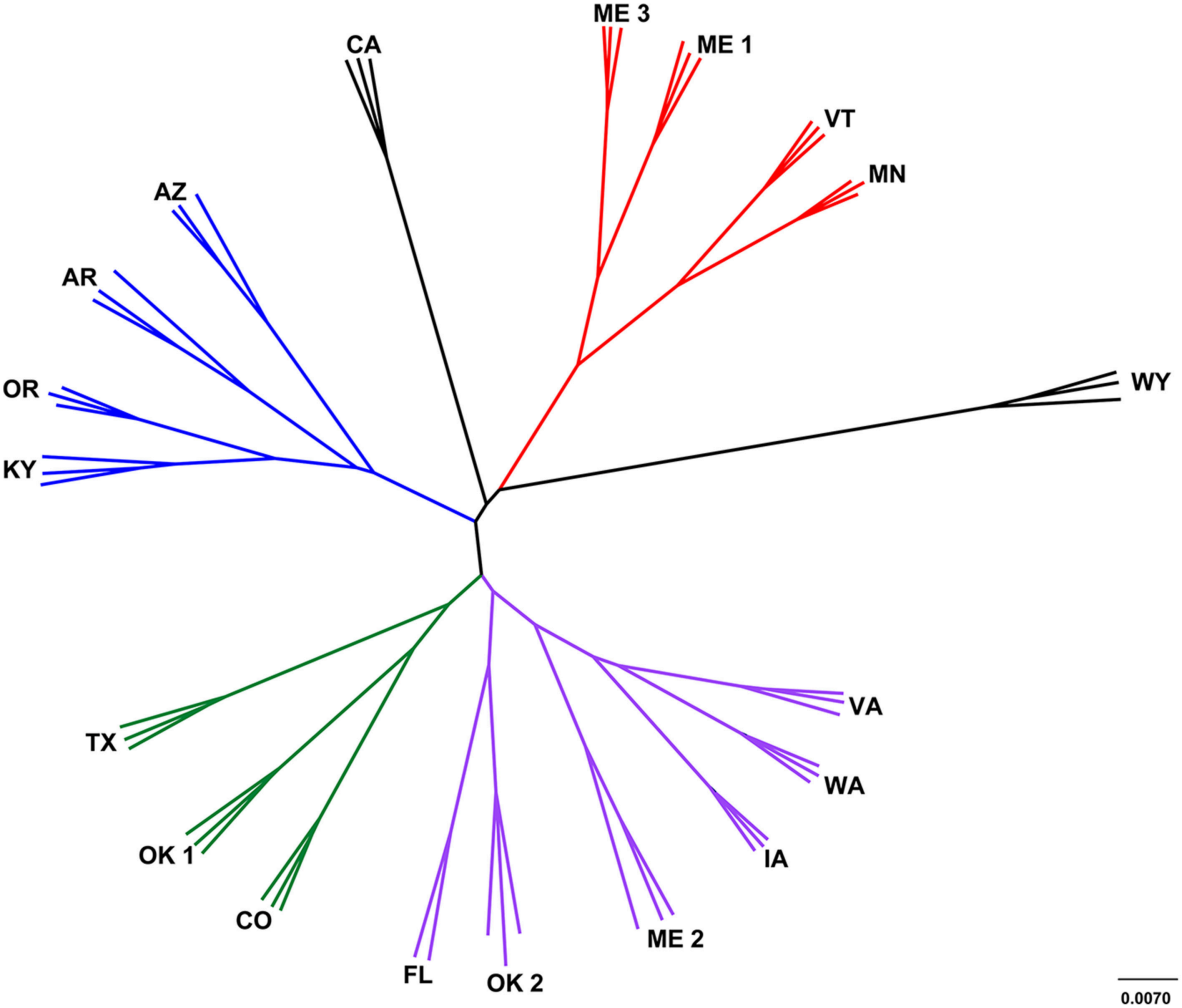
**Cluster analysis of landfill leachates represented by a jackknifed UPGMA tree**. Clade A (green) contained TX, OK1, and CO; clade B (red) contained ME1, ME3, VT and MN; clade C (purple) contained VA, WA, IA, ME2, OK2, and FL; and clade D (blue) contained AZ, AR, OR, and KY Two landfills, CA and WY (black) were outliers to the other four distinct clades.

**Figure 4:**
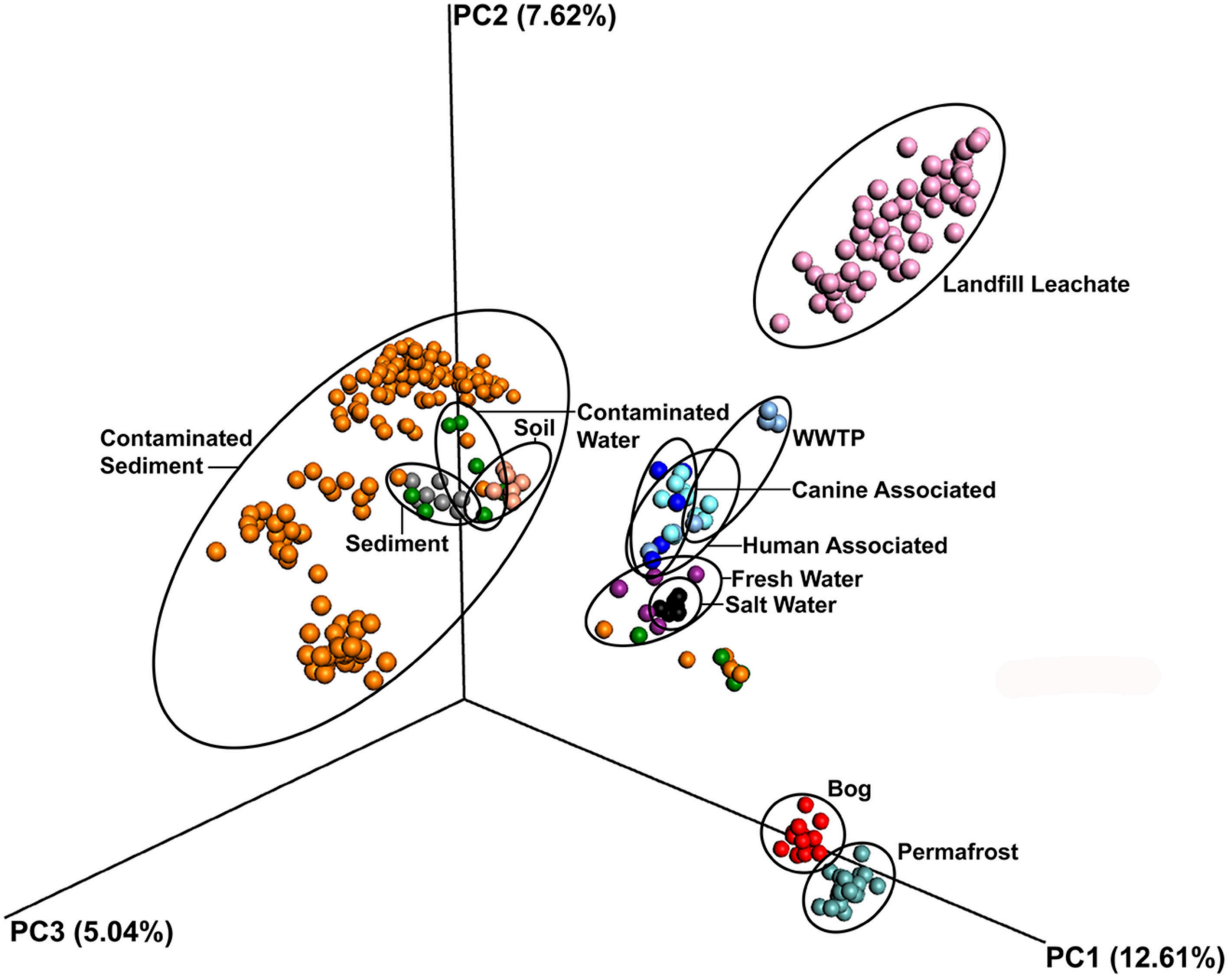
**Principal component ordination analysis of microbial communities from diverse environments based on an unweighted UniFrac distance matrix, showing the distinct grouping of communities in landfill leachate sampled for this study from all others**. Samples are colored by source.

The leachate microbial assemblages formed four significantly distinct clades (*p* < 0.001, *R*^2^ = 0.586), with two outlying landfills (CA and WY) based on the phylogenetic similarity between samples (weighted and unweighted UniFrac) using the UPGMA method (Figure 3). All landfill leachate microbial assemblages were unique among all other ecosystems included in the meta-analysis, based on a Principle Coordinate Analysis (Figure 4). The results of the meta-analysis suggested that the selective forces within the landfill were unique.

Results of multiple PERMANOVA analyses suggested that while all parameters tested *a priori* were significant, the separation (indicated by the *R*^2^ value) was poor (Table 2). Testing the prediction that landfills would cluster by rainfall amounts produced a significant *p*-value (*p* = 0.001), however the *R*^2^ value was low (0.073) indicating poor separation of the communities by this variable alone. Instead, samples separated more clearly by geographic region (*p* = 0.01, *R*^2^ = 0.36). Of the other 159 geochemical and environmental parameters available, *post-hoc* tests suggested that 125 were significant (*p* ≤ 0.05), yet only 16 *R*^2^ values were above 0.10 (Table S6). The only CECs with an *R*^2^ above 0.10 were camphor and chloroxylenol.

**Table 2:** **PERMANOVA results of regional or operational parameters assumed to be significant *a priori***.

**Parameter**	***p*-value**	***R*^2^ Value**
Region	0.001	0.357
Rainfall	0.001	0.073
Age (Class)	0.001	0.117
Waste (Mton/Yr)	0.001	0.070
Leachate produced (Mgal/Yr)	0.003	0.055
Waste dissolution time	0.006	0.052

The exploratory statistical method Bio-Env was used to identify combinations of variables that best explained the distribution of weighted UniFrac distances among the landfill leachate microbial assemblages. The concentrations of barium and chloride, and the mean evapotranspiration time were identified as explanatory variables with the highest correlation ρ (rho) value of 0.568 (Table S11). A similar, but slightly lower ρ-value of 0.533 was obtained that included the above variables, as well as age of waste and the number of detected household chemicals (Table S11; Masoner et al., 2014).

The microbial assemblages found in the landfill leachate samples were compared among each other and against the microbiomes of sediments, soils, fresh water, salt water, bogs, permafrost, humans, and canines. Ordination based on unweighted UniFrac distances from a total of 65697 OTUs revealed that microbial assemblages from landfill leachates formed a significantly (*p* < 0.001, *R*^2^ = 0.294) distinct and distant clade (Figure 4). Likewise, the microbial assemblages from soil, sediment, marine or freshwater, bogs and permafrost, and mammals largely clustered to the exclusion of one another.

## Discussion

Much of what is produced and used by humans is eventually disposed of in a landfill along with its resident microorganisms. Landfills are, therefore, a potential final resting place for much of the human-derived (i.e., the built) environment and its biodegradation. The degradation of this waste releases greenhouse gases and solubilizes a vast array of chemicals including CECs (Masoner et al., 2014).

Landfills are home to diverse assemblages of bacteria and archaea capable of a broad range of biodegradation activities (Mori et al., 2003; Gomez et al., 2011; Lu et al., 2012). Consequently, microbial metabolism is a primary driver of the degradation of MSW in landfills, resulting in the release of Non-Volatile Dissolved Organic Carbon (NVDOC), CECs, and landfill gases. These microbial communities are difficult to study, partly due to the high physical and chemical heterogeneity of a landfill. Previous studies have attempted to address this heterogeneity by collecting a large number of samples at a single landfill (Palmisano and Barlaz, 1996) or sampling a single location within a landfill to better understand the stratification and therefore the age of waste (Suflita et al., 1992). Both of these approaches can be costly, time-prohibitive, and still represent only a small cross section of the broad distribution of deposited waste. Other studies of landfills have investigated landfill cores or cover soils (Henneberger et al., 2014). These studies did not view landfills in a greater context, or as a single potentially novel biome. Unlike previous studies investigating the microbial communities of landfills (McDonald et al., 2010; Mouser et al., 2010; Lu et al., 2012), the study described here is the first to investigate a large number of landfills through sampling of leachate and high throughput sequencing of 16S rRNA gene libraries. Landfill leachate flows by the path of least resistance through the landfill and arguably is more representative of the broader microbiological communities in a large number of landfills.

The microbiomes characterized in the sampled landfill leachates grouped into four primary clades (Figure 3). Leachate microbiomes in clade A were composed predominantly of members of the Clostridiales. Notably, no single family within the Clostridiales was dominant. Instead, a large number of different members of the Clostridiales appeared to inhabit the landfills within clade A. The Ruminococcaceae were the most abundant single family (3–6%), members of which are associated with the degradation of cellulose, a common carbon source within landfills, and within mammalian guts (Hungate, 1966). In addition, members of the Peptococcaceae, Campylobacterales, and Bacteroidales have all been correlated with the degradation of hydrocarbons in multiple environments (Lyles et al., 2013), similar to the CECs detected within the landfills studied (Masoner et al., 2014).

The microbiomes in leachate samples within clade B were unique in the abundance of members of the order Campylobacterales. Almost half of the sequences in one landfill in clade B grouped within a single OTU within the Campylobacteraceae that was most closely related to the genus *Arcobacter*. Members of the genus *Arcobacter* have been discovered previously in MSW leachate plumes (Huang et al., 2005; Tian et al., 2005), wastewater effluent streams (Santos et al., 2009; Merga et al., 2014), and are commonly associated with pathogenesis (Vandenberg et al., 2004). To the exclusion of the Campylobacteraceae, the abundance of members of the order Pseudomonadales was also a notable trait of leachate microbiomes in this clade. Detected members of the Pseudomonadales were most closely related to the genera *Pseudomonas* and *Acinetobacter*, both of which are capable of mineralizing many of the recalcitrant aromatic compounds present in MSW (Beal and Betts, 2000). In addition, landfills within this clade were less diverse than any other grouping of landfills. This was due to the abundance of the Campylobacteraceae, possibly due to the intrusion of dissolved oxygen in the leachates of this clade, although this prediction was not tested during the chemical study of the landfills.

Landfill leachate microbiomes within clade C were unique in their relative dissimilarity to all other landfills (Figure 3). Their microbiomes were phylogenetically diverse, and included members of the Chlorobi and within one landfill (FL), OP9. Detected members of the Chlorobi were closely related to uncultured lineages of the class Ignavibacteriales, which contains organisms capable of fermentation under slightly thermophilic conditions (Iino et al., 2010; Podosokorskaya et al., 2013). Single-cell genomic and metagenomic approaches have identified members of candidate division OP9 as being capable of anaerobic, fermentative metabolism of sugars resulting in the production of hydrogen, acetate, and ethanol (Dodsworth et al., 2013). The OP9 genomes also contained genes for putative glucohydrolase and endonuclease enzymes that could be used for the catabolism of (hemi) celluloses. While (hemi) cellulose was not specifically assayed for, FL was deplete in low molecular weight carbon sources such as acetate, yet high in NVDOC relative to the other sampled landfills.

Landfills grouping within clade D contained a larger population of the candidate division OP3, Methylococcales, and the Desulfobacterales. Except for AR, these landfills displayed a high concentration of barium (AZ, KY, and OR) and a correspondingly low concentration of sulfate (Table S4). Members of the candidate division OP3 belong to the PVC superphylum. Metagenomic and single-cell genome studies have revealed that members of OP3 share characteristics with the Deltaproteobacteria, including the ability to reduce sulfate (Glöckner et al., 2010). Sulfate reduction through the dissolution of barite, which is found in clays, drilling muds, paint, paper, cloth, and rubber, could produce the high concentration of barium observed (Ulrich et al., 2003).

Outlying landfills (WY and CA) were also notable in the abundance of the Euryarchaeota and unclassified OTUs. For instance, a single unclassified OTU represented between 6 and 13% of detected taxa at the WY landfill that was most closely related to mitochondrial sequence from Oomycetes. These organisms are similar in body plan to fungi and capable of degrading a broad diversity of carbohydrates (Horner et al., 2012), but the specific role they may play in landfills is unknown (Lockhart et al., 2006). Other unclassified members of the community were related to clones found in subsurface waters, or oxygen minimum zones (Divya et al., 2011), which along with the abundance of Deltaproteobacteria and OP3 suggest that the landfills of clade D are oxygen depleted. The landfill CA contained the highest percentage of methanogenic archaea of any landfill leachate sampled, along with the high relative abundance (5.7–6.9%) of Thermoplasmatales (composite relative abundance for all Thermoplasmatales OTUs in Table S8). Members of the Thermoplasmatales are commonly acidophilic thermophiles that are not known to be methanogenic, although recent research has shown that a candidate genus “*Methanomethylophilus*,” within the order Thermoplasmatales is capable of methanogenesis (Paul et al., 2012; Yadav et al., 2015). Based on their abundance in landfills in this study and others (Yadav et al., 2015), landfills appear to be an ecosystem that favors these novel methanogens.

The findings described in this study suggest that landfills are a source of considerable bacterial and archaeal diversity. A functional gene array-based survey of groundwater impacted by landfill leachate however, suggested that diversity decreases with proximity to landfill sites (Lu et al., 2012). One explanation for the findings of Lu et al. would be that genes from the abundant, deeply branching bacterial lineages detected in this study may have little homology to the probes on their gene array. Alternatively, the microorganisms found in landfill leachates may not be capable of surviving in the more dilute groundwater environment, and the diversity of microorganisms in the impacted groundwater may be negatively impacted by the leachate chemistry, thus explaining the reduction in organismal diversity. Metagenomic characterization of landfills could provide some insight toward the functional genes of the unclassified, but abundant populations across many landfill leachate systems.

More than 100 geochemical and environmental parameters were tested *post-hoc* in this study. Such a large number of tested parameters caused difficulty by likely resulting in spurious correlations. To reduce the effect of such a large number of pairwise comparisons, we employed FDR correction of *p*-values. A complex set of CECs were present in the leachate from most of the 19 landfills in this study, with rainfall previously implicated as a predictor of CEC abundance (Masoner et al., 2014). The majority of CECs and rainfall also had an effect on microbial community structure and composition (*p* < 0.05), but the low *R*^2^ values suggested that their influence on the distribution and relative abundance of the microbial population was minimal. This finding would reject the prediction that rainfall strongly influences the overall distribution and relative abundance of microbial communities in landfill leachate. Instead, the single most powerful influence on landfill community distribution and composition appeared to be geographic region, suggesting that numerous regional factors play a role in establishing the members of the landfill leachate microbial community. Examples of contributing regional factors might include climate conditions, the composition of deposited wastes, and the geochemistry of soils used for entombment.

Much like the varied input to the landfills, a combination of parameters instead of any single input may have the greatest impact on the overall observed diversity of landfills. In combination, the concentration of barium and chloride, the rate of evapotranspiration, and the age of waste were variables that best explained the distribution of microbial composition across all landfill leachates sampled. Only a small number of CECs detected could be considered as producing a significant effect and more than a minimal correlation. It is possible that this is due to the reduced statistical power of running such a large number of tests. An alternative explanation for this would be that the resident microbial community at each site is either unaffected by many of the CECs, does not interact with them in any meaningful way, or both.

This study affirms that landfills and their leachates foster a unique microbiome, essentially distinct from any ecosystem previously investigated (Figure 4). When using a conservative OTU picking method, such as UPARSE, over 4000 OTUs were detected across the characterized microbiomes. The large number and phylogenetic diversity of OTUs is likely due to the large number of available niches linked, in part, to the diversity of possible soluble electron acceptors and oxidizable substrates present within the leachate of each landfill (Masoner et al., 2014). These compounds are dispersed throughout the depositional structure of a landfill. Over time, however, endogenous water production, water infiltration, and rainfall allow the chemistry and biology to potentially comingle, and produce the unique geochemical composition of landfill leachate. While not all deposited materials in a landfill will solubilize, landfill leachate still contains a large quantity of dissolved organic components (Nanny and Ratasuk, 2002). Over time scales spanning hours (initially) to seasons and decades, there is significant variation in the availability of electron acceptors and carbon sources (Cozzarelli et al., 2011). The result of this variability is an environment under constantly changing selective pressures, which could also account for the high evenness and species (OTU) richness seen across the leachate microbial assemblages. The landfill leachate, therefore, represents a rich, diverse “seed bank” (Konopka et al., 2014) potentially able to respond to the extensive and varying chemical inputs a landfill receives. This study did not attempt to study landfill leachates over time, and therefore cannot specifically address the temporal influence potentially driving the microbial community of this unique biome.

The daily deposition of heterogeneous materials into landfills and their progressive biodegradation create a unique selective landscape responsible for the novel biodiversity found in their leachates. This biodiversity is an untapped source of genomic, metabolic, and biochemical innovation with great potential benefit to bioremediation efforts, bioindustrial processes, and the discovery of new natural products. The work presented here provides the foundation for subsequent efforts that should focus on establishing direct links between observed unclassified taxa and their metabolic capabilities. Directed and novel cultivation-based approaches could lead to the enrichment of consortia or isolation of individual microorganisms capable of degrading targeted compounds (Stevenson et al., 2004; Nichols et al., 2010). Additionally, metagenomics and single-cell genomics approaches have proven invaluable in characterizing the putative metabolic capacity of yet uncultured organisms (Dodsworth et al., 2013; McLean et al., 2013; Stamps et al., 2014; Nobu et al., 2015). Landfills and their leachates are an essential component in modeling the interaction between humanity and the biosphere, and therefore should be included in the ongoing, coordinated efforts to understand and harness the capabilities of Earth's microbial ecosystems (Alivisatos et al., 2015).

## Author contributions

BS contributed in the experimental design, conducting experiments, data analysis, and writing the manuscript. CL participated in the experimental design, conducting experiments, and writing the manuscript. JS contributed to the experimental design and writing the manuscript. JM, IC, and DK contributed to the experimental design, data interpretation, coordinated and/or conducted sampling, and writing the manuscript. BS contributed in the experimental design, conducting experiments, data analysis and interpretation, and writing the manuscript.

## Funding

This project was supported by the USGS Toxics Substances Hydrology Program and USGS Oklahoma Water Science Center under grant #G12AC20148.

### Conflict of interest statement

The authors declare that the research was conducted in the absence of any commercial or financial relationships that could be construed as a potential conflict of interest.

## Supplementary material

Supplementary Information **Details of PCR and Barcoding PCR conditions**.

Figure S1 **Phylogenetic tree based on the non-redundant tree (SILVA r123 NR99) within the phylogenetic software package ARB, which is composed of all unclassified OTUs from landfill leachates and closely related sequences**. Taxonomic groupings are labeled and delineated with brackets. Scale bar represents a nucleotide dissimilarity of 0.10.

Table S1 **Volume (mL) filtered and concentration (ng/μL) of DNA recovered from each leachate sample**.

Table S2 **QIIME-compatible mapping file used for comparison of landfill leachates**.

Table S3 **QIIME-compatible mapping file used for meta-analyses of microbial communities**.

Table S4 **Environmental variables for leachates used in PERMANOVA analyses**.

Table S5 **Concentrations of CECs (ng/mL) for leachates used in PERMANOVA analyses**.

Table S6 **Results from PERMANOVA analyses of all^a^*post-hoc* tests of environmental variables and CEC concentrations**.

Table S7 **Diversity indices for sampled leachate microbiomes**.

Table S8 **Relative abundance of OTUs detected in each landfill leachate sample**.

Table S9 **Absolute abundance of OTUs detected in each landfill leachate sample**.

Table S10 **Taxonomy of OTUs found in the landfill leachate core microbiome**.

Table S11 **Results of BIOENV analysis**.
