# Supplementary material for: Municipal Solid Waste Landfills Harbor Distinct Microbiomes
Source: Front Microbiol. 2016 Apr 20;7:534. doi: 10.3389/fmicb.2016.00534 (PMC4837139; doi:10.3389/fmicb.2016.00534)

Supplementary Information

PCR conditions

For all samples, an initial 50µL PCR consisting of 1x DreamTaq PCR master mix (ThermoFisher Scientific, Waltham, MA, USA), 0.1 µM of each primer, and 5-10 µL of 1:10 dilutions of DNA extractions was carried out. Cycling conditions were as follows: initial denaturation of 95˚C for 2m, followed by 30 cycles of 95˚C for 30s, 52˚C for 45s, 72˚C for 45s, ending with a final extension of 72˚C for 10m, and a hold forever at 10˚C. Subsequent barcoding reactions followed the same cycling conditions, but for only 6 cycles. A complete list of barcodes used can be found as a mapping file in Table S2.

Barcoding PCR reaction

The forward primer M13L-519F was appended with a unique sequence (5’- **GTA AAA CGA CGG CCA G -3’**) at the 5’ end of the primer that is complementary to the sequence found on all barcodes (5’- NNN NNN NNN NNN **GTA AAA CGA CGG CCA G -3’**). This allows for the use of a limited cycle PCR for barcoding, and re-use of barcodes with multiple primer sets. A pictorial representation of the approach is below.


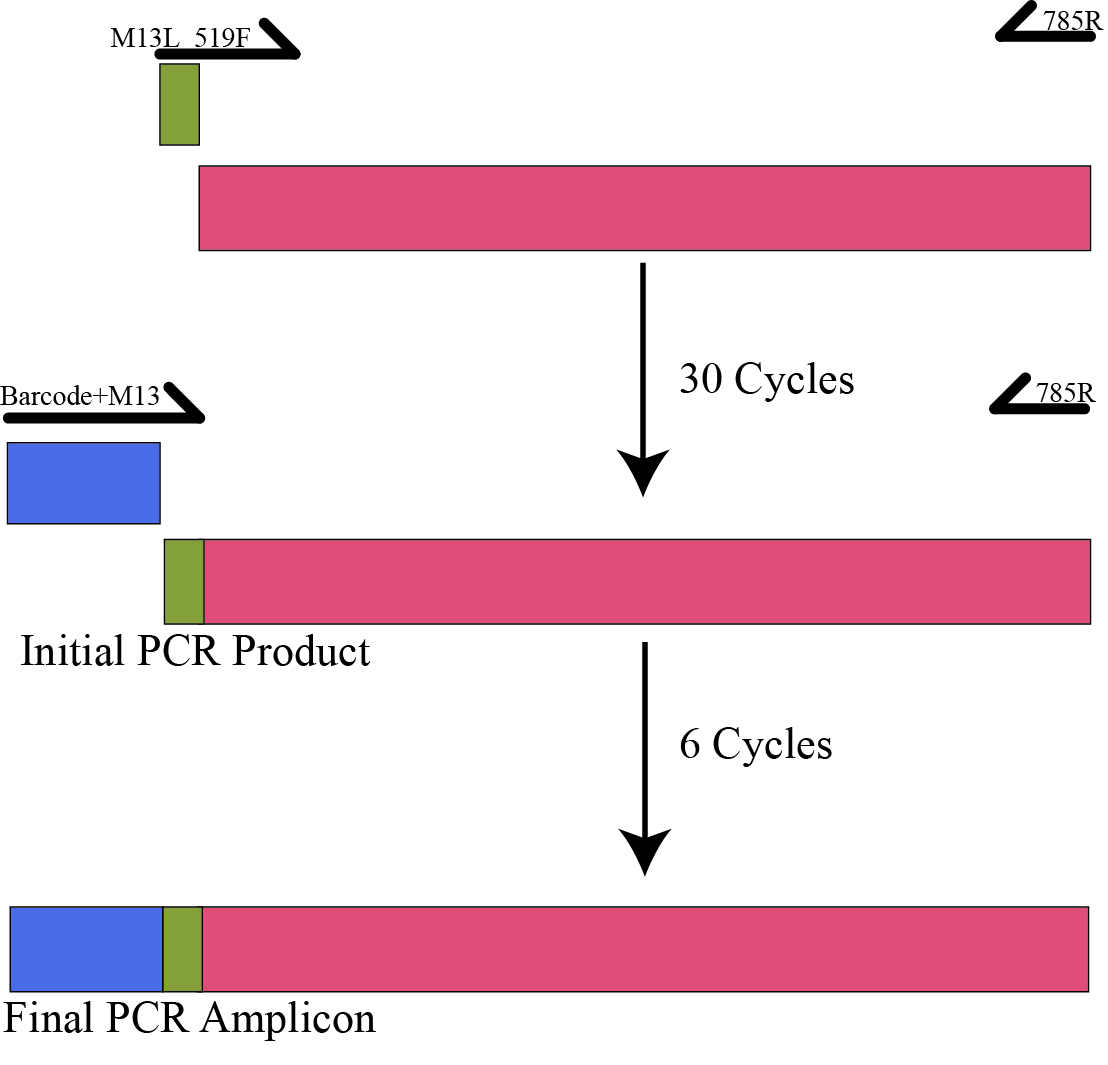

Supplement: Supplementary Information — Details of PCR and Barcoding PCR conditions. [file DataSheet1.DOCX]
