# Supplementary figures and images for: Municipal Solid Waste Landfills Harbor Distinct Microbiomes
Source: Front Microbiol. 2016 Apr 20;7:534. doi: 10.3389/fmicb.2016.00534 (PMC4837139; doi:10.3389/fmicb.2016.00534)

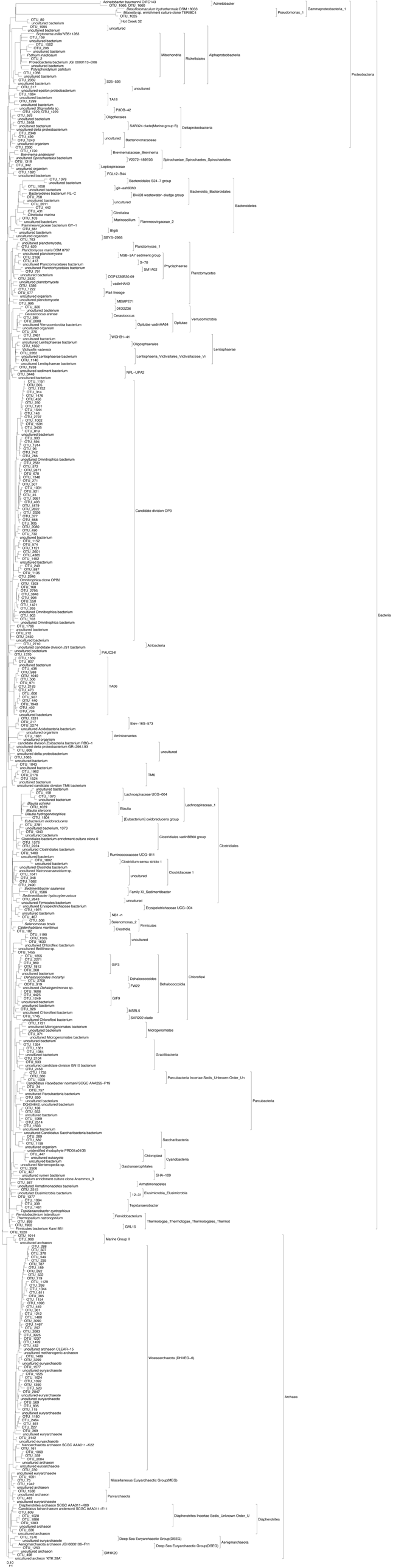

Supplement: Figure S1 — Phylogenetic tree based on the non-redundant tree (SILVA r123 NR99) within the phylogenetic software package ARB, which is composed of all unclassified OTUs from landfill leachates and closely related sequences. Taxonomic groupings are labeled and delineated with brackets. Scale bar represents a nucleotide dissimilarity of 0.10. [file Image1.PDF]
