# Supplementary material for: Municipal Solid Waste Landfills Harbor Distinct Microbiomes
Source: Front Microbiol. 2016 Apr 20;7:534. doi: 10.3389/fmicb.2016.00534 (PMC4837139; doi:10.3389/fmicb.2016.00534)
Supplement: Table S6 — Results from PERMANOVA analyses of allapost-hoc tests of environmental variables and CEC concentrations. [file Table6.DOCX]

**Table S6:** Results from PERMANOVA analyses of all^a^ post hoc tests of environmental variables and CEC concentrations.

| **Category** |  | **SumsOfSqs** | **MeanSqs** | **F.Model** | **R^2^** | **Pr(>F)** | **FDR^b^** |
| --- | --- | --- | --- | --- | --- | --- | --- |
| 4.cumylphenol |  | 0.24041 | 0.240405 | 4.5192 | 0.077 | 0.001 | 0.012 |
| aciclovir |  | 0.18221 | 0.182208 | 3.3572 | 0.059 | 0.001 | 0.012 |
| Active |  | 0.30404 | 0.304041 | 5.8449 | 0.098 | 0.001 | 0.012 |
| Ag |  | 0.36495 | 0.36495 | 7.1713 | 0.117 | 0.001 | 0.012 |
| Al |  | 0.26131 | 0.261313 | 4.9483 | 0.084 | 0.001 | 0.012 |
| albuterol |  | 0.21994 | 0.21994 | 4.1052 | 0.071 | 0.001 | 0.012 |
| amphetamine |  | 0.2624 | 0.262401 | 4.9707 | 0.084 | 0.001 | 0.012 |
| antipyrene |  | 0.17484 | 0.174836 | 3.2133 | 0.056 | 0.001 | 0.012 |
| atenolol |  | 0.22032 | 0.22032 | 4.1129 | 0.071 | 0.001 | 0.012 |
| B |  | 0.29401 | 0.294012 | 5.632 | 0.094 | 0.001 | 0.012 |
| Ba |  | 0.32658 | 0.32658 | 6.3289 | 0.105 | 0.001 | 0.012 |
| bisphenol.a |  | 0.2642 | 0.264199 | 5.008 | 0.085 | 0.001 | 0.012 |
| camphor |  | 0.35683 | 0.35683 | 6.9912 | 0.115 | 0.001 | 0.012 |
| carisoprodol |  | 0.20323 | 0.20323 | 3.7716 | 0.065 | 0.001 | 0.012 |
| Cd |  | 0.38935 | 0.38935 | 7.7193 | 0.125 | 0.001 | 0.012 |
| Chloroxylenol |  | 0.40561 | 0.40561 | 8.0901 | 0.130 | 0.001 | 0.012 |
| cholesterol |  | 0.2536 | 0.253598 | 4.7892 | 0.081 | 0.001 | 0.012 |
| cis.androsterone |  | 0.22283 | 0.22283 | 4.1633 | 0.072 | 0.001 | 0.012 |
| Cl |  | 0.33285 | 0.33285 | 6.465 | 0.107 | 0.001 | 0.012 |
| Clade by UPGMA clustering |  | 1.5979 | 0.31959 | 10.547 | 0.513 | 0.001 | 0.012 |
| Co |  | 0.27848 | 0.278485 | 5.3054 | 0.089 | 0.001 | 0.012 |
| cotinine |  | 0.29284 | 0.292837 | 5.6072 | 0.094 | 0.001 | 0.012 |
| Cr |  | 0.31624 | 0.31624 | 6.106 | 0.102 | 0.001 | 0.012 |
| Cu |  | 0.29212 | 0.292121 | 5.592 | 0.094 | 0.001 | 0.012 |
| desvenlafaxine |  | 0.23351 | 0.233514 | 4.3792 | 0.075 | 0.001 | 0.012 |
| dextromethorphan |  | 0.18011 | 0.180113 | 3.3162 | 0.058 | 0.001 | 0.012 |
| estrone |  | 0.1776 | 0.1776 | 3.2671 | 0.057 | 0.001 | 0.012 |
| famotidine |  | 0.18912 | 0.189123 | 3.4928 | 0.061 | 0.001 | 0.012 |
| fexofenadine |  | 0.23504 | 0.235039 | 4.4101 | 0.076 | 0.001 | 0.012 |
| fluconazole |  | 0.24974 | 0.249744 | 4.7101 | 0.080 | 0.001 | 0.012 |
| Formate |  | 0.20707 | 0.20707 | 3.8479 | 0.067 | 0.001 | 0.012 |
| HCO3 |  | 0.18252 | 0.182518 | 3.3633 | 0.059 | 0.001 | 0.012 |
| HormonesSteriodsCount |  | 0.24986 | 0.249858 | 4.7124 | 0.080 | 0.001 | 0.012 |
| ibuprofen |  | 0.27915 | 0.279152 | 5.3193 | 0.090 | 0.001 | 0.012 |
| IndustrialWastePercent |  | 0.24262 | 0.242622 | 4.5644 | 0.078 | 0.001 | 0.012 |
| K |  | 0.39506 | 0.39506 | 7.8491 | 0.127 | 0.001 | 0.012 |
| lamivudine |  | 0.19179 | 0.191789 | 3.5453 | 0.062 | 0.001 | 0.012 |
| lidocaine |  | 0.24243 | 0.242432 | 4.5605 | 0.078 | 0.001 | 0.012 |
| meprobamate |  | 0.25679 | 0.25679 | 4.8549 | 0.082 | 0.001 | 0.012 |
| metaxalone |  | 0.17497 | 0.174972 | 3.2159 | 0.056 | 0.001 | 0.012 |
| Mg |  | 0.26594 | 0.265937 | 5.044 | 0.085 | 0.001 | 0.012 |
| Mo |  | 0.26238 | 0.262376 | 4.9702 | 0.084 | 0.001 | 0.012 |
| MunWastePercent |  | 0.21472 | 0.214722 | 4.0006 | 0.069 | 0.001 | 0.012 |
| N.desmethyldiltiazem |  | 0.25767 | 0.25767 | 4.873 | 0.083 | 0.001 | 0.012 |
| N.N.diethyltoluamide..DEET. |  | 0.25823 | 0.258228 | 4.8846 | 0.083 | 0.001 | 0.012 |
| Na |  | 0.35139 | 0.35139 | 6.8711 | 0.113 | 0.001 | 0.012 |
| NH3 |  | 0.37804 | 0.37804 | 7.464 | 0.121 | 0.001 | 0.012 |
| NH4 |  | 0.37793 | 0.37793 | 7.4618 | 0.121 | 0.001 | 0.012 |
| Ni |  | 0.26741 | 0.267413 | 5.0746 | 0.086 | 0.001 | 0.012 |
| nicotine |  | 0.23007 | 0.230074 | 4.3095 | 0.074 | 0.001 | 0.012 |
| NonprescriptionPharmsCount |  | 0.30305 | 0.303047 | 5.8238 | 0.097 | 0.001 | 0.012 |
| NVDOC |  | 0.21547 | 0.215468 | 4.0156 | 0.069 | 0.001 | 0.012 |
| oxycodone |  | 0.16229 | 0.16229 | 2.97 | 0.052 | 0.001 | 0.012 |
| pentachlorophenol |  | 0.2082 | 0.208197 | 3.8704 | 0.067 | 0.001 | 0.012 |
| PesticideChemicalsCount |  | 0.19413 | 0.194132 | 3.5915 | 0.062 | 0.001 | 0.012 |
| pH |  | 0.20692 | 0.206916 | 3.8448 | 0.066 | 0.001 | 0.012 |
| phenol |  | 0.21246 | 0.212457 | 3.9553 | 0.068 | 0.001 | 0.012 |
| piperonyl.butoxide |  | 0.23289 | 0.23289 | 4.3665 | 0.075 | 0.001 | 0.012 |
| PlantAnimalSterolsCount |  | 0.35715 | 0.35715 | 6.9982 | 0.115 | 0.001 | 0.012 |
| PO42 |  | 0.21616 | 0.216163 | 4.0295 | 0.069 | 0.001 | 0.012 |
| Pyruvate |  | 0.19064 | 0.190642 | 3.5227 | 0.061 | 0.001 | 0.012 |
| Rb |  | 0.29627 | 0.296274 | 5.6799 | 0.095 | 0.001 | 0.012 |
| Sb |  | 0.33527 | 0.33527 | 6.5177 | 0.108 | 0.001 | 0.012 |
| Se |  | 0.28208 | 0.282083 | 5.3807 | 0.091 | 0.001 | 0.012 |
| Si |  | 0.30552 | 0.305517 | 5.8764 | 0.098 | 0.001 | 0.012 |
| skatol |  | 0.26114 | 0.261139 | 4.9447 | 0.084 | 0.001 | 0.012 |
| Sn |  | 0.25799 | 0.25799 | 4.8796 | 0.083 | 0.001 | 0.012 |
| SO42 |  | 0.19519 | 0.195193 | 3.6124 | 0.063 | 0.001 | 0.012 |
| Specific Conductance |  | 0.32338 | 0.32338 | 6.2598 | 0.104 | 0.001 | 0.012 |
| Sr |  | 0.31554 | 0.315542 | 6.091 | 0.101 | 0.001 | 0.012 |
| thiabendazole |  | 0.17585 | 0.175853 | 3.2331 | 0.056 | 0.001 | 0.012 |
| tramadol |  | 0.23887 | 0.23887 | 4.4879 | 0.077 | 0.001 | 0.012 |
| tri.dichlorisopropyl.phosphate |  | 0.18751 | 0.187508 | 3.4611 | 0.060 | 0.001 | 0.012 |
| U |  | 0.21471 | 0.214708 | 4.0004 | 0.069 | 0.001 | 0.012 |
| V |  | 0.3079 | 0.307895 | 5.9272 | 0.099 | 0.001 | 0.012 |
| WasteType |  | 0.23855 | 0.238552 | 4.4815 | 0.077 | 0.001 | 0.012 |
| wwSludgePercent |  | 0.2128 | 0.212803 | 3.9623 | 0.068 | 0.001 | 0.012 |
| 10.hydroxy.amitriptyline |  | 0.18467 | 0.184674 | 3.4055 | 0.059 | 0.002 | 0.019 |
| 2.methylnapthalene |  | 0.16395 | 0.163949 | 3.0021 | 0.053 | 0.002 | 0.019 |
| acetophenone |  | 0.17858 | 0.17858 | 3.2863 | 0.057 | 0.002 | 0.019 |
| As |  | 0.17898 | 0.178982 | 3.2941 | 0.058 | 0.002 | 0.019 |
| beta.sitosterol |  | 0.18161 | 0.181607 | 3.3454 | 0.058 | 0.002 | 0.019 |
| Br |  | 0.18673 | 0.18673 | 3.4459 | 0.060 | 0.002 | 0.019 |
| carbamazepine |  | 0.18051 | 0.180508 | 3.3239 | 0.058 | 0.002 | 0.019 |
| cimetidine |  | 0.21648 | 0.216482 | 4.0359 | 0.070 | 0.002 | 0.019 |
| fenofibrate |  | 0.17602 | 0.176017 | 3.2363 | 0.057 | 0.002 | 0.019 |
| lorazepam |  | 0.17842 | 0.178421 | 3.2832 | 0.057 | 0.002 | 0.019 |
| metformin |  | 0.18334 | 0.183344 | 3.3794 | 0.059 | 0.002 | 0.019 |
| methadone |  | 0.19773 | 0.197726 | 3.6625 | 0.064 | 0.002 | 0.019 |
| oxazepam |  | 0.18916 | 0.189157 | 3.4935 | 0.061 | 0.002 | 0.019 |
| Propionate |  | 0.1981 | 0.1981 | 3.6699 | 0.064 | 0.002 | 0.019 |
| pseudoephedrine |  | 0.18584 | 0.185835 | 3.4283 | 0.060 | 0.002 | 0.019 |
| Zn |  | 0.24209 | 0.242095 | 4.5536 | 0.078 | 0.002 | 0.019 |
| acetaminophen |  | 0.17388 | 0.173877 | 3.1946 | 0.056 | 0.003 | 0.026 |
| Butyrate |  | 0.17061 | 0.170612 | 3.1311 | 0.055 | 0.003 | 0.026 |
| Cs |  | 0.19142 | 0.191424 | 3.5381 | 0.061 | 0.003 | 0.026 |
| morphine |  | 0.15866 | 0.15867 | 2.9001 | 0.051 | 0.003 | 0.026 |
| nadolol |  | 0.15019 | 0.150186 | 2.7373 | 0.048 | 0.003 | 0.026 |
| rac.cis.N.desmethylsertraline |  | 0.15768 | 0.157679 | 2.8811 | 0.051 | 0.003 | 0.026 |
| stigmastanol |  | 0.19773 | 0.197728 | 3.6625 | 0.064 | 0.003 | 0.026 |
| W |  | 0.17631 | 0.176313 | 3.2421 | 0.057 | 0.003 | 0.026 |
| benzophenone |  | 0.14567 | 0.14567 | 2.6509 | 0.047 | 0.004 | 0.032 |
| benzotriazole.methyl.1H |  | 0.15134 | 0.151344 | 2.7595 | 0.049 | 0.004 | 0.032 |
| diphenhydramine |  | 0.16329 | 0.163292 | 2.9894 | 0.052 | 0.004 | 0.032 |
| isopropylbenzene |  | 0.16893 | 0.16893 | 3.0986 | 0.054 | 0.004 | 0.032 |
| Li |  | 0.15301 | 0.15301 | 2.7914 | 0.049 | 0.004 | 0.032 |
| loperamide |  | 0.16734 | 0.167338 | 3.0676 | 0.054 | 0.004 | 0.032 |
| para.cresol |  | 0.18225 | 0.182245 | 3.3579 | 0.059 | 0.004 | 0.032 |
| phenytoin |  | 0.17294 | 0.172938 | 3.1763 | 0.056 | 0.004 | 0.032 |
| prednisolone |  | 0.14893 | 0.14893 | 2.7133 | 0.048 | 0.004 | 0.032 |
| sulfamethizole |  | 0.17555 | 0.175547 | 3.2271 | 0.056 | 0.004 | 0.032 |
| temazepam |  | 0.1574 | 0.157398 | 2.8757 | 0.051 | 0.004 | 0.032 |
| venlafaxine |  | 0.16311 | 0.16311 | 2.9858 | 0.052 | 0.004 | 0.032 |
| caffeine |  | 0.14608 | 0.146081 | 2.6588 | 0.047 | 0.005 | 0.037 |
| loratadine |  | 0.14967 | 0.149669 | 2.7274 | 0.048 | 0.005 | 0.037 |
| methocarbamol |  | 0.16612 | 0.166121 | 3.0441 | 0.053 | 0.005 | 0.037 |
| metoprolol |  | 0.1661 | 0.1661 | 3.0437 | 0.053 | 0.005 | 0.037 |
| Pb |  | 0.17808 | 0.178076 | 3.2764 | 0.057 | 0.005 | 0.037 |
| triphenyl.phosphate |  | 0.14334 | 0.143336 | 2.6064 | 0.046 | 0.005 | 0.037 |
| Ca |  | 0.14103 | 0.14103 | 2.5625 | 0.045 | 0.006 | 0.044 |
| sulfadimethoxine |  | 0.15396 | 0.153956 | 2.8096 | 0.049 | 0.006 | 0.044 |
| bupropion |  | 0.15692 | 0.156916 | 2.8664 | 0.050 | 0.007 | 0.050 |
| estriol |  | 0.14591 | 0.145912 | 2.6555 | 0.047 | 0.007 | 0.050 |
| glipizide |  | 0.15413 | 0.154135 | 2.813 | 0.050 | 0.007 | 0.050 |
| tri.2.chloroethyl.phosphate |  | 0.13949 | 0.139485 | 2.5331 | 0.045 | 0.008 | 0.057 |
| naphthalene |  | 0.14731 | 0.14731 | 2.6822 | 0.047 | 0.009 | 0.064 |
| CollectionSource |  | 0.14135 | 0.141349 | 2.5686 | 0.045 | 0.010 | 0.069 |
| para.nonylphenol.total |  | 0.13581 | 0.135807 | 2.4632 | 0.044 | 0.010 | 0.069 |
| warfarin |  | 0.14089 | 0.140891 | 2.5598 | 0.045 | 0.010 | 0.069 |
| Anthracene |  | 0.13644 | 0.136442 | 2.4753 | 0.044 | 0.011 | 0.075 |
| pentoxifylline |  | 0.13415 | 0.134151 | 2.4319 | 0.043 | 0.011 | 0.075 |
| Acetate |  | 0.1418 | 0.141802 | 2.5772 | 0.046 | 0.012 | 0.080 |
| HouseHoldandPesticidesCount |  | 0.14636 | 0.146358 | 2.6641 | 0.047 | 0.012 | 0.080 |
| 1.methylnapthalene |  | 0.12968 | 0.129684 | 2.3474 | 0.042 | 0.013 | 0.085 |
| ConstDebrisPercent |  | 0.14386 | 0.14386 | 2.6164 | 0.046 | 0.013 | 0.085 |
| triclosan |  | 0.13268 | 0.132676 | 2.4039 | 0.043 | 0.013 | 0.085 |
| HousholdChemicalsCount |  | 0.13185 | 0.131847 | 2.3883 | 0.042 | 0.014 | 0.090 |
| Lactate |  | 0.13527 | 0.135274 | 2.4531 | 0.043 | 0.014 | 0.090 |
| Mn |  | 0.13289 | 0.132885 | 2.4079 | 0.043 | 0.014 | 0.090 |
| Fe |  | 0.12612 | 0.126117 | 2.2801 | 0.041 | 0.016 | 0.102 |
| 1.4.dichlorobenzene |  | 0.13052 | 0.130518 | 2.3631 | 0.042 | 0.017 | 0.107 |
| paraxanthine |  | 0.12193 | 0.12193 | 2.2013 | 0.039 | 0.018 | 0.113 |
| 17.beta.estradiol |  | 0.1296 | 0.129605 | 2.3459 | 0.042 | 0.019 | 0.118 |
| 3.beta.coprostanol |  | 0.12333 | 0.123328 | 2.2276 | 0.040 | 0.021 | 0.129 |
| theophylline |  | 0.12355 | 0.12355 | 2.2318 | 0.040 | 0.021 | 0.129 |
| MunicipalOrPrivate |  | 0.11457 | 0.114573 | 2.0634 | 0.037 | 0.030 | 0.183 |
| pentobarbital |  | 0.11058 | 0.11058 | 1.9889 | 0.036 | 0.031 | 0.188 |
| IndustrialChemicalsCount |  | 0.11128 | 0.111279 | 2.0019 | 0.036 | 0.032 | 0.193 |
| 4.tert.octylphenol |  | 0.10313 | 0.103128 | 1.8502 | 0.033 | 0.045 | 0.269 |
| glyburide |  | 0.09725 | 0.097254 | 1.7414 | 0.031 | 0.049 | 0.291 |
| tributylphosphate |  | 0.1003 | 0.100299 | 1.7978 | 0.032 | 0.061 | 0.360 |
| menthol |  | 0.09606 | 0.09606 | 1.7194 | 0.031 | 0.087 | 0.510 |
| diethylhexyl.phthalate |  | 0.09391 | 0.093915 | 1.6798 | 0.030 | 0.088 | 0.513 |
| OilFieldWasteAccepted |  | 0.008742 | 0.0087424 | 1.6437 | 0.031 | 0.099 | 0.573 |
| NO3 |  | 0.08851 | 0.088506 | 1.5802 | 0.028 | 0.107 | 0.615 |
| 4.tert.Octylphenol.monoethoxylate |  | 0.08108 | 0.081085 | 1.4442 | 0.026 | 0.164 | 0.937 |
| 4.Nonylphenol.diethoxylate |  | 0.06916 | 0.069164 | 1.227 | 0.022 | 0.228 | 1.000 |
| diethyl.phthalate |  | 0.05845 | 0.058449 | 1.0333 | 0.019 | 0.364 | 1.000 |

^a^ Any category present in Masoner et. al 2014 where detectable quantities were present in less than two landfills were omitted.

^b^ FDR correction performed by the method discussed in Benjamini and Yekutieli 2001.
