# Supplementary material for: Municipal Solid Waste Landfills Harbor Distinct Microbiomes
Source: Front Microbiol. 2016 Apr 20;7:534. doi: 10.3389/fmicb.2016.00534 (PMC4837139; doi:10.3389/fmicb.2016.00534)
Supplement: Table S7 — Diversity indices for sampled leachate microbiomes. [file Table7.DOCX]

| Landfill |  | ACE | SD |  | PD | SD |  | Obs OTUs | SD |  | *E_H_* | SD |
| --- | --- | --- | --- | --- | --- | --- | --- | --- | --- | --- | --- | --- |
| WY |  | 2120.57 | 30.27 |  | 26.50 | 0.30 |  | 1129.90 | 7.71 |  | 0.81 | 0.01 |
| TX |  | 2201.61 | 20.06 |  | 30.18 | 0.31 |  | 1348.10 | 21.41 |  | 0.88 | 0.01 |
| OK1 |  | 2313.46 | 140.19 |  | 27.95 | 0.63 |  | 1243.80 | 62.12 |  | 0.82 | 0.01 |
| CO |  | 2103.21 | 50.74 |  | 25.06 | 0.22 |  | 1175.60 | 20.25 |  | 0.83 | 0.01 |
| CA |  | 2187.60 | 129.92 |  | 27.09 | 0.62 |  | 1274.00 | 42.62 |  | 0.85 | 0.00 |
| KY |  | 2125.89 | 13.06 |  | 23.33 | 0.28 |  | 1122.40 | 33.92 |  | 0.79 | 0.03 |
| OR |  | 2357.85 | 59.99 |  | 25.10 | 0.39 |  | 1249.70 | 23.21 |  | 0.84 | 0.01 |
| AR |  | 2457.38 | 34.72 |  | 28.42 | 0.37 |  | 1314.80 | 20.60 |  | 0.80 | 0.01 |
| AZ |  | 1951.81 | 141.13 |  | 22.01 | 0.60 |  | 1002.40 | 31.91 |  | 0.77 | 0.01 |
| MN |  | 2254.88 | 65.94 |  | 23.63 | 0.11 |  | 1127.00 | 16.62 |  | 0.76 | 0.00 |
| VT |  | 2016.19 | 53.22 |  | 22.17 | 0.47 |  | 1059.70 | 26.15 |  | 0.76 | 0.01 |
| VA |  | 2209.67 | 69.30 |  | 25.22 | 0.54 |  | 1249.90 | 32.65 |  | 0.86 | 0.01 |
| WA |  | 2249.92 | 60.29 |  | 24.97 | 0.93 |  | 1332.40 | 45.92 |  | 0.87 | 0.00 |
| IA |  | 2292.00 | 46.22 |  | 26.58 | 0.44 |  | 1302.80 | 12.44 |  | 0.83 | 0.00 |
| ME2 |  | 2407.07 | 147.15 |  | 26.12 | 1.30 |  | 1298.70 | 87.05 |  | 0.82 | 0.05 |
| OK2 |  | 2074.55 | 107.08 |  | 25.06 | 0.87 |  | 1103.80 | 85.82 |  | 0.80 | 0.03 |
| FL |  | 2029.30 | 122.61 |  | 23.37 | 0.69 |  | 1090.90 | 21.00 |  | 0.78 | 0.02 |
| ME3 |  | 2161.68 | 88.21 |  | 23.30 | 0.50 |  | 1090.90 | 35.91 |  | 0.76 | 0.01 |
| ME1 |  | 1999.23 | 54.02 |  | 21.50 | 0.53 |  | 926.20 | 37.15 |  | 0.64 | 0.02 |

**Table S7:** Diversity indices for sampled leachate microbiomes.

^a^ Median values and standard deviation (SD) for abundance-based coverage estimation (ACE), Faith’s Phylogenetic Diversity (PD), number of observed OTUs (Obs OTUs), and Shannon’s equitability index (*E_H_*) from replicate samples (n=3).
